# Novel Calibration Algorithm for a Three-Axis Strapdown Magnetometer

**DOI:** 10.3390/s140508485

**Published:** 2014-05-14

**Authors:** Yan Xia Liu, Xi Sheng Li, Xiao Juan Zhang, Yi Bo Feng

**Affiliations:** 1 College of Automation, Beijing Union University, Beijing 100101, China; 2 School of Automation and Electrical Engineering, University of Science and Technology Beijing, Beijing 100083, China; E-Mails: lxs@ustb.edu.cn (X.S.L.); zxjjianghan@163.com (X.J.Z.); feng_yibo@163.com (Y.B.F.)

**Keywords:** constant intersection angle assumption, ellipsoid fitting, restricted least squares solution, rotation matrix

## Abstract

A complete error calibration model with 12 independent parameters is established by analyzing the three-axis magnetometer error mechanism. The said model conforms to an ellipsoid restriction, the parameters of the ellipsoid equation are estimated, and the ellipsoid coefficient matrix is derived. However, the calibration matrix cannot be determined completely, as there are fewer ellipsoid parameters than calibration model parameters. Mathematically, the calibration matrix derived from the ellipsoid coefficient matrix by a different matrix decomposition method is not unique, and there exists an unknown rotation matrix *R* between them. This paper puts forward a constant intersection angle method (angles between the geomagnetic field and gravitational field are fixed) to estimate *R*. The Tikhonov method is adopted to solve the problem that rounding errors or other errors may seriously affect the calculation results of *R* when the condition number of the matrix is very large. The geomagnetic field vector and heading error are further corrected by *R*. The constant intersection angle method is convenient and practical, as it is free from any additional calibration procedure or coordinate transformation. In addition, the simulation experiment indicates that the heading error declines from ±1° calibrated by classical ellipsoid fitting to ±0.2° calibrated by a constant intersection angle method, and the signal-to-noise ratio is 50 dB. The actual experiment exhibits that the heading error is further corrected from ±0.8° calibrated by the classical ellipsoid fitting to ±0.3° calibrated by a constant intersection angle method.

## 1. Introduction

Accurate measurement of the geomagnetic field has been widely applied in geophysical research, space magnetic measurement, military defense, mineral resources exploration, and drilling practice [1]. Common magnetometers include a helium optical-pumping magnetometer, a proton magnetometer, a three-axis flux gate magnetometer, and an anisotropic magnetoresistive magnetometer. The former two magnetometers could only be used to measure geomagnetic field magnitude, whereas the latter two magnetometers, belonging to the vector magnetometer type, are the most common magnetic sensors in an attitude and heading reference system (AHRS), which is characterized by low cost and high reliability. However, the magnetometer is full of inevitable errors such as zero deviation, sensitivity errors, non-orthogonal errors, misalignment errors, measurement noise, hard iron errors, and soft iron errors, and consequently there are large errors between the measurement results and actual geomagnetic field vector. As a result, calibration and error compensation should be conducted before the application. The calibration of magnetometers can be divided into the geomagnetic domain calibration and the heading domain calibration. The heading domain calibration only calibrates the heading error of the magnetic compass composed of the magnetometer, but each axis output of the magnetometer has not been calibrated. The geomagnetic domain calibration directly calibrates the output of each axis of the magnetometer. The magnetometer output after calibration can not only calculate the accurate heading angle $\left(\alpha =\tan^{-1}(H_{v}^{n}/H_{x}^{n})\right)$ but also can be used in geomagnetic navigation for providing rich component information.

The “two-step” algorithm directly calibrates the magnetometer error without an external heading reference. The principle of the two-step algorithm is that when the magnitude of the true geomagnetic field is invariable at the calibration spot, the ideal locus of the three-axis magnetometer output lies on a spherical surface, whereas the actual locus of the magnetometer measurement outcome lies on an ellipsoid under the disturbance of error. The first step of the two-step algorithm transforms the non-linear observation equation based on the square of the magnetic field magnitude into a linear equation of the new unknown parameter by a mathematical transformation and acquires the unknown parameter by a batch least squares solution. In the second step, the biases and the scale factors can be derived from an inverse analytical solution [2], but the non-orthogonal and misalignment errors cannot be calibrated because of the error model, which includes only zero deviation and sensitivity error. The iterative batch least squares method is used in [3] to obtain the ellipsoid parameter unbiased estimation, from which the initial conditions can be acquired by the aforementioned two-step algorithm. The algorithm possesses consistent convergence, and the posterior covariance could be used as a metric for the compensation effect. The “extension of the two-step” algorithm improves the two-step calibration algorithm by compensating for the non-orthogonal error [4]. However, as the assumed sensor *x*-axis coincides with the body *x*-axis (it is also possible that none of the three axes coincides in reality), the misalignment error cannot be calibrated.

It is proposed in literature [5] that there are only nine independent parameters in the fitting ellipsoid model. Failing to clarify the 12 parameters in the calibration model mathematically could also be interpreted to mean that there is more than one method to decompose the ellipsoid coefficient matrix into two orthogonal matrixes (error parameter matrix). According to matrix theory, an orthogonal rotation matrix (indicating the rotation transform in three-dimensional space) exists between matrixes, resulting from different decomposition methods. In other words, magnetometer data can be compensated for sensor errors and the presence of magnetic distortions by mapping an ellipsoid of data to a sphere; however, the rotation of the sphere is unknown [5,6]. The essence of this difference discloses the failure to clarify the three-axis misalignment error. It has been shown in [7] that the magnetometer measurement noise is zero-mean Gaussian white noise, but the noise of the square of the magnetometer output is not zero mean, and ordinary least square (OLS) estimation is an inconsistent biased estimation. Adaptive least square (ALS) is utilized to obtain a consistent unbiased estimator of ellipsoid parameters because of the continuous adjustment procedure to the OLS cost function. This adjustment requires a known noise variance. Consistent estimation to an unknown noise variance, however, can be realized [8]. ALS improves the accuracy of the ellipsoid fitting, but the calibration parameters derived by eigen decomposition are not always true because of the different matrix decomposition methods mentioned above. In addition, the coefficient matrix of the ellipsoid fitting could also be used to derive the error parameter matrix from singular value decomposition [9]. However, the error model of this method approximates a symmetric matrix, which contains only nine undetermined coefficients; thus, the application of this method has limitations. In [10] a geomagnetic field vector and some auxiliary vector dot products are choosen as the constants to exam the effect of the rotation matrix in rectifying and compensating the error. This method does not require additional calibration, and the compensation accuracy depends on the choice of auxiliary vector. When the auxiliary vector is nearly parallel with the geomagnetic vector, the worst compensation result is obtained, whereas the best result is obtained when the former vector is perpendicular to the latter vector. Because the auxiliary vector is a constant vector of the earth coordinate system, it should be converted into components of the body coordinate system in a mathematical operation. Maximum likelihood estimation is used in the error parameter fitting, and misalignment error requires additional independent calibration for identification [11].

The transition matrix defined in [12] between the sensor coordinate system and body system contains six unknown parameters. This matrix completely describes the non-orthogonal error and misalignment error and adopts the “three-step” algorithm in magnetometer pre-calibration before flight, without reference to any direction information. The misalignment rotation matrix can be derived from the condition that the vertical component of the geomagnetic field remains unchanged while rotating the magnetometer respectively around the *x*-axis, *y*-axis, and *z*-axis in the geoid. It is proposed in [13] that the rotation matrix of the misalignment error could be obtained when the sensor rotates around an axis, and the projection of the magnetic field output is invariable, which lies on the plane that is perpendicular to the said axis. This method is more valuable in engineering as it is unnecessary to keep leveling. This paper is based on the constant intersection angle between the geomagnetic field vector and gravitation field vector [14], without additional independent calibration and coordinate system conversion, and seeks to derive a rotation matrix more conveniently, furthering the compensation of magnetic field measurement error and heading error.

## 2. Error Modeling

Because of the influence of the manufacturing processes and environment, a three-axis magnetometer has manufacturing, installation and environmental errors. The manufacturing error contains a zero-deviation *H*_0_ and sensitivity error *K*_s_; installation error includes non-orthogonal error *k*_nor_ and misalignment error $T_{\text{m}}^{\text{b}}$; environmental error includes the soft iron error *C*_s_ and hard iron error *H*_p_. On account of all the errors listed above, the relationship between the true geomagnetic field *H*_e_ and the three-axis magnetometer output *H*_m_ is demonstrated in Equation (1), in which *H_n_* indicates the Gaussian white noise is zero-mean and its standard deviation is σ:

(1) $$H_{\text{m}}=K_{\text{s}}\cdot K_{\text{nor}}\cdot T_{\text{m}}^{\text{b}}\cdot C_{\text{s}}(H_{\text{e}}+H_{\text{p}})+H_{0}+H_{\text{n}}=M\cdot (H_{\text{e}}+H_{\text{p}})+H_{0}+H_{n}$$

The different types of errors are specifically shown in Subsections 2.1–2.6.

### 2.1. Sensitivity Error

Sensitivity represents the proportional relationship between the magnetometer input and output. Sensitivity error results from the inconsistency between the amplification of the measuring electronic circuit and its nominal value, which can be expressed as:

(2) $$K_{\text{s}}=\text{diag}(K_{\text{s}x}\cdot K_{\text{s}y}\cdot K_{\text{s}z})$$

If sensitivity is the only error that affects the sensor, the Equation (2) refers to the measurement *H*_m_ = *K*_s_ · *H*_e_.

### 2.2. Zero Deviation

There is often a small voltage bias in the sensor output signal, even when there is no magnetic intensity. In such conditions, the sensor produces non-zero output, which can be expressed as:

(3) $$H_{0}={\left[\begin{array}{ccc} H_{0x} & H_{0y} & H_{0z} \end{array}\right]}^{T}$$

If zero deviation is the only error that affects the sensor, the Equation (3) refers to the measurement *H*_m_ = *H*_e_ + *H*_0_.

### 2.3. Non-Orthogonal Error

**Figure 1.**
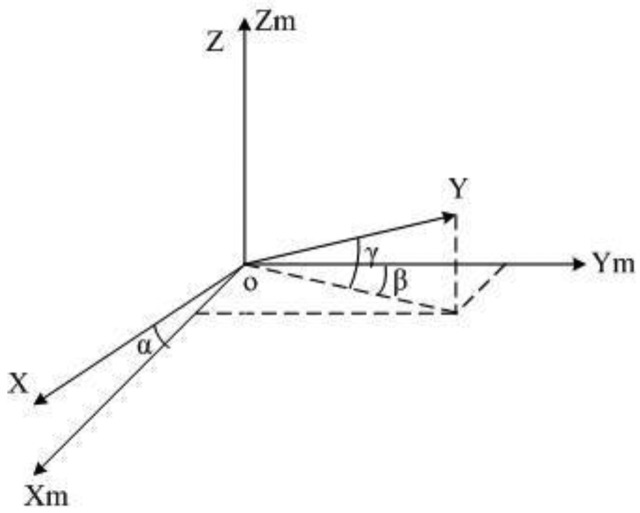
Sensor coordinate system non-orthogonal error.

Non-orthogonal error is caused by the limitation of machining, which leads the *x*-, *y*- and *z*-axes to not being completely orthogonal to each other. This is shown in Figure 1, assuming that the *z*-axis of the sensor completely coincides with the *z*-axis of the orthogonal coordinate system *T*_m_:

(4) $$K_{\text{nor}}=\left[\begin{array}{ccc} \cos\alpha & 0 & \sin\alpha \\ \sin\beta \text{cos}\gamma & \cos\beta \text{cos}\gamma & \text{sin}\gamma \\ 0 & 0 & 1 \end{array}\;\right]$$

If non-orthogonal error is the only error that affects the sensor, Equation (4) refers to the measurement *H*_m_ = *K*_nor_ · *H*_e_.

### 2.4. Misalignment Error

**Figure 2.**
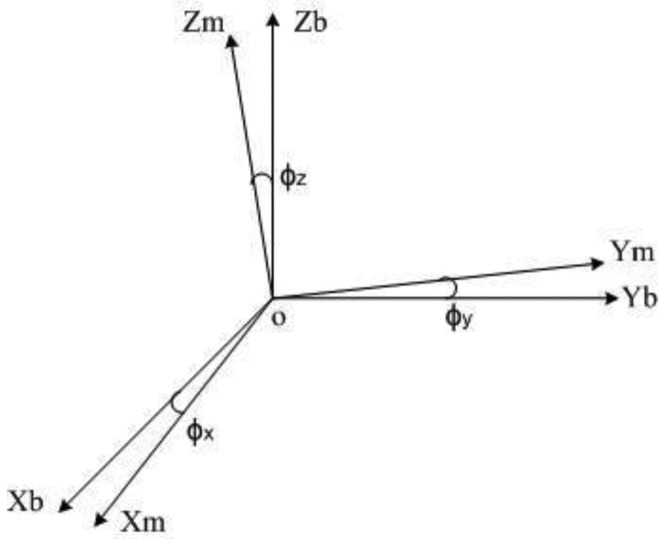
Body orthogonal coordinate system and virtual orthogonal coordinate system.

During the installation, the sensor *x*-axis may not totally coincide with the body vertical axis, as illustrated in Figure 2. The resultant transition matrix (rotation matrix) $T_{\text{m}}^{\text{b}}$ exists between the virtual orthogonal coordinate system *T*_m_ and the body orthogonal coordinate system $T_{\text{m}}^{\text{b}}$, which is equivalent to a slight angle generated from rotations of the sensor around the *x*-axis, *y*-axis and *z*-axis, respectively:

(5) $$T_{\text{m}}^{\text{b}}=\left[\begin{array}{lll} t_{xx} & t_{xy} & t_{xz}\\ t_{yx} & t_{yy} & t_{yz}\\ t_{zx} & t_{zy} & t_{zz} \end{array}\right]$$

If misalignment is the only error that affects the sensor, the Equation (2) refers to the measurement $H_{\text{m}}=T_{\text{m}}^{\text{b}}\cdot H_{\text{e}}\cdot H$.

### 2.5. Soft Iron Errors

Ferromagnetic materials produce an inductive magnetic field under the influence of a geomagnetic field and the induced electric current around the sensor. It also varies with the attitude of the magnetometer and the position in the magnetic field, which can be expressed as:

(6) $$C_{\text{s}}=\left[\begin{array}{lll} a_{xx} & a_{xy} & a_{xz}\\ a_{yx} & a_{yy} & a_{yz}\\ a_{zx} & a_{zy} & a_{zz} \end{array}\right]$$

The *a_ij_* terms represent the effective soft iron coefficients and are the constants of proportionality between the magnetic field applied to soft iron and the resulting induced magnetic field [3]. For example, *a_xy_* represents the effective coefficient relating the field generated in the *x*-direction in response to an applied field in the *y*-direction, where the effective soft iron coefficients appears insignificant when *i* ≠ *j*. Mathematically, the soft iron error for principal diagonal elements is equivalent to the sensitivity error; and non-diagonal elements correspond to the non-orthogonal error and misalignment error. If soft iron is the only error that affects the sensor, Equation (2) takes to the measurement *H*_m_ = *C*_s_ · *H*_e_.

### 2.6. Hard Iron Errors

This type of error results from permanent magnets and magnetic hysteresis, that is, remanence of magnetized iron materials. Mathematically, this error is equivalent to zero-deviation:

(7) $$H_{\text{p}}={\left[\begin{array}{ccc} H_{\text{p}x} & H_{\text{p}y} & H_{\text{p}z} \end{array}\right]}^{\text{T}}$$

If hard iron is the only error that affects the sensor, Equation (2) refers to the measurement *H*_m_ = *H*_e_ + *H*_p_.

Ignoring the measurement noise in Equation (1), the geomagnetic field vector *H*_e_ can be derived as:

(8) $$H_{\text{e}}=G\cdot (H_{\text{m}}-H_{0})-H_{\text{p}}$$

in which $G=M^{-1}=\left[\begin{array}{ccc} g_{11} & g_{12} & g_{13}\\ g_{21} & g_{22} & g_{23}\\ g_{31} & g_{32} & g_{33} \end{array}\right]$.

## 3. Classical Ellipsoid Assumption Algorithm

When an ideal magnetometer rotates in the calibration spot, the geomagnetic field magnitude remains constant, and the output locus of the three-axis magnetometer is a sphere. In contrast, a non-ideal magnetometer output locus would be an ellipsoid because of the influence of error. The square of *H*_e_ is derived from Equation (8), and sorted into the quadratic form of the surface:

(9) $$H_{\text{m}}^{T}\frac{G^{T}G}{{\left\|H_{\text{e}}\right\|}^{2}}H_{\text{m}}-2\frac{H_{0}^{T}G^{T}G+{\text{H}}_{\text{p}}^{T}G}{{\left\|H_{\text{e}}\right\|}^{2}}H_{\text{m}}+\frac{{\text{H}}_{0}^{T}G^{T}GH_{0}+2{\text{H}}_{\text{p}}^{T}GH_{0}+{\text{H}}_{\text{p}}^{T}H_{\text{p}}}{{\left\|H_{\text{e}}\right\|}^{2}}=1$$

Equation (9) is rewritten as a general form of the quadratic surface equation:

(10) $$F\left(\xi ,\;H_{\text{m}}\right)=aH_{\text{m}x}^{2}+bH_{\text{m}x}H_{\text{m}y}+cH_{\text{m}y}^{2}+dH_{\text{m}x}H_{\text{m}z}+eH_{\text{m}y}H_{\text{m}z}+fH_{\text{m}z}^{2}+pH_{\text{m}x}+qH_{\text{m}y}+rH_{\text{m}z}+s=0$$

where ξ = (*a, b, c, d, e, f, p, q, r, s*)*^T^*.

Generally, non-diagonal elements of the soft iron matrix *C*_s_, the non-orthogonal error and misalignment error of the soft iron error, are minuscle, therefore, the error matrix *M* is strictly diagonally dominant [5]. Therefore *G^T^G* is also strictly diagonally dominant, belonging to a positive-definite real symmetric matrix, and Equation (9) is a quadratic ellipsoid equation, then for convenience, Equation (9) can be written as:

(11) $$X^{T}AX-2X_{0}^{T}AX+X_{0}^{T}AX_{0}=1$$

where, $A=\left[\begin{array}{ccc} a & b/2 & d/2\\ b/2 & c & e/2\\ d/2 & e/2 & f \end{array}\right]$ is the coefficient matrix after ellipsoid fitting, and $X_{0}=-A^{-1}\left[\begin{array}{c} p\\ q\\ r \end{array}\right]$ are the spherical center coordinates. The objective of magnetometer calibration is to conduct ellipsoid fitting based on the set of *N* measurement data, deriving the coefficient matrix *A* and spherical center coordinates *X*_0_.

The principle of ellipsoid fitting is finding an ideal ellipsoid where the sum of squares of the algebraic distances calculated from the measured data point to the ellipsoid is minimized [15], which is expressed as Equation (12) or (13):

(12) $$\underset{\xi \in R^{10}}{\min}\sum_{i=1}^{N}{\left\|F(\xi ,H_{\text{mi}})\right\|}^{2}$$

(13) $$\underset{\xi \in R^{10}}{\min}\xi^{T}D^{T}D\xi$$

where: $D=\left[\begin{array}{rrrrrr} H_{\text{m}x1}^{2} & H_{\text{m}x1}H_{\text{m}y1} & H_{\text{m}y1}^{2}H_{\text{m}x1}H_{\text{m}z1}H_{\text{m}y1}H_{\text{m}z1} & H_{\text{m}z1}^{2}H_{\text{m}x1} & H_{\text{m}y1}H_{\text{m}z1} & 1\\ & \vdots & \vdots & & & \vdots \\ & H_{\text{m}xN}^{2} & \cdots & & & 1 \end{array}\right]$.

In order to assure that the fitting result is an ellipsoid, Nikos contends that parameter σ should guarantee that matrix *A* in Equation (10) is positive definite matrix or negative matrix [16]. This constraint is analyzed in literature [16], which states that the equality constraint 4*ab* − *b*^2^ = 1 can be imposed for the convenience of calculation, which would not lose its generality and can be expressed in the matrix form:

(14) $$\xi^{T}C\xi =1$$

where $C=\left[\begin{array}{cc} C_{1} & 0_{3\times 7}\\ 0_{7\times 3} & 0_{7\times 7} \end{array}\right]$ and $C_{1}=\left[\begin{array}{ccc} 0 & 0 & 2\\ 0 & -1 & 0\\ 2 & 0 & 0 \end{array}\right]$.

By introducing a Lagrange multiplier λ, we can obtain the simultaneous equation from Equation (13) and Equation (14) as follows:

(15) $$G(\xi )=\xi^{T}D^{T}D\xi +\lambda (1-\sigma^{T}C\xi )$$

In order to obtain the extremum under constraints, the first-order derivative could be derived from Equation (15), and Equation (16) could be obtained when the first-order derivative of Equation (15) is set to zero:

(16) $$D^{T}D\xi =\lambda C\xi$$

Therefore, the extremum under an ellipsoid constraint could be obtained:

(17) $$\{\begin{array}{c} D^{T}D\xi =\lambda C\xi \\ \xi^{T}C\xi =1 \end{array}$$

It has been proved in [17] that only one of the generalized eigenvalues in Equation (16) is positive, and its eigenvector is the best ellipsoid fitting parameter ξ. The ellipsoid coefficient matrix *A* and spherical center coordinate *X*_0_ is derived from further calculation. It could be concluded from the comparison between Equation (9) and Equation (10) that:

(18) $$\{\begin{array}{c} G^{T}G={\left\|H_{\text{e}}\right\|}^{2}\times A\\ H_{\text{p}}=G(X_{0}-H_{0}) \end{array}$$

‖*H*_e_‖ is not necessarily the actual magnitude of the geomagnetic field because its magnitude only affects the components on the *x*-axis and *y*-axis, whereas the heading hinges on the proportion of the geomagnetic field projection on the x-coordinate and y-coordinate [9]. Introducing *H*_p_ = *G*(*X*_0_ − *H*_0_) to Equation (8), we obtain *H*_e_ = *G*(*H*_m_ − *H*_0_) − *G*(*X*_0_ − *H*_0_) = *G*(*H*_m_ − *X*_0_). Therefore, it is not necessary to use *H*_0_. The 3 × 3 matrix *G* combining scale factors, non-orthogonal errors, misalignments, and soft iron disturbances is not strict symmetrical, including nine independent parameters, and there are three combined biases in the error model. The total number of error parameters should be 12.

Therefore, the key to deriving calibration matrix *G* is to decompose the ellipsoid coefficient matrix into two orthogonal matrixes. However, the calibration matrix cannot be clarified, as the orthogonal matrix resulting from a different decomposition is not exactly the same. Because *G* is also a positive definite matrix, the decomposition results *G*1 and *G*2 only differ in an orthogonal rotation matrix *R*, which is equivalent to a fixed rotating angle in the body coordinate system between the geomagnetic field vector after compensation and the true geomagnetic field vector [5].

This paper provides a constant intersection angle method to solve the rotation matrix *R*. On the basis of this rotation matrix, the matrix *G* resulting from orthogonal decomposition could be transformed into the calibration matrix that is needed. The calibrated geomagnetic field could be obtained by solving Equation (8) and Equation (18) simultaneously:

(19) $$H_{\text{e}}=Q\cdot (H_{\text{m}}-X_{0})$$

where *Q* = *R* · *G*.

## 4. Constant Intersection Angle Method

Similar to the classical ellipsoid fitting method, the constant intersection angle method also requires calibration in the same location in order to satisfy the condition that the magnitude and direction of the geomagnetic field and gravitation field are invariant. The intersection angle between the geomagnetic field vector and gravitation field vector at the same measurement spot is constant [14], and the dot product of magnetic field vector H_e_ and gravity acceleration vector *A*_g_, which ‖*H*_e_‖ · *A*_g_ · cosθ, is invariable and can be calculated from the model of geomagnetic field and gravitation field. In the said equation, θ is the intersection angle between the geomagnetic field vector and gravitation field vector, and its value can be interpreted to have approximately the same effect as ‖*H*_e_‖ in Equation (18). Therefore, different values of θ would only affect the magnitude of the geomagnetic field after calibration rather than the heading solution. In addition, the acceleration coordinate system is also the body coordinate system as the accelerometer is fixed on the body and keeps moving with the body. Thus the intersection angle between the geomagnetic field vector *H*_e_ and gravitation field vector *A*_g_ at the same measurement spot is constant, even if there is no transformation into a geodetic coordinate system.

### 4.1. Rotation Matrix Solution

To begin, calibration matrix *G* is obtained by a singular value decomposition of the positive matrix ‖*H*_e_‖_2_ *A* generated from Equation (18). Then, *H*_c_ can be derived as below, by introducing *G* and *X*_0_ into Equation (19) and preliminarily calibrating the geomagnetic field:

(20) $$H_{\text{c}}=G\cdot (H_{\text{m}}-X_{0})$$

where the gravity acceleration vector is $A_{\text{g}}=\left[\begin{array}{c} A_{\text{g}x}\\ A_{\text{g}y}\\ A_{\text{g}z} \end{array}\right]$, the magnetic field vector is *H*_e_ = *R* · *H*_c_, and *R* as the undetermined rotation matrix can be represented as $R=\left[\begin{array}{ccc} R_{11} & R_{12} & R_{13}\\ R_{21} & R_{22} & R_{23}\\ R_{31} & R_{32} & R_{33} \end{array}\right]$. Additionally, the dot product of the magnetic field vector and the gravity acceleration vector could is described as:

(21) $${A_{\text{g}y}}^{T}\cdot H_{\text{e}}={\left[\begin{array}{c} A_{\text{g}x}\\ A_{\text{g}y}\\ A_{\text{g}z} \end{array}\right]}^{T}\cdot \left[\begin{array}{lll} R_{11} & R_{12} & R_{13}\\ R_{21} & R_{22} & R_{23}\\ R_{31} & R_{32} & R_{33} \end{array}\right]\cdot \left[\begin{array}{c} H_{\text{c}x}\\ H_{\text{c}y}\\ H_{\text{c}z} \end{array}\right]=\left\|H_{\text{e}}\right\|\cdot \left\|A_{\text{g}}\right\|\cdot \cos\theta =\text{const}$$

Recasting Equation (21) as a linear combination of a changing input vector and an estimation parameter vector, Equation (22) is obtained:

(22) $$\text{const}={\left[\begin{array}{l} A_{\text{g}x}H_{\text{c}x}\\ A_{\text{g}x}H_{\text{c}y}\\ A_{\text{g}x}H_{\text{c}z}\\ A_{\text{g}y}H_{\text{c}x}\\ A_{\text{g}y}H_{\text{c}y}\\ A_{\text{g}y}H_{\text{c}z}\\ A_{\text{g}z}H_{\text{c}x}\\ A_{\text{g}z}H_{\text{c}y}\\ A_{\text{g}z}H_{\text{c}z} \end{array}\right]}^{\text{T}}\cdot \left[\begin{array}{l} R_{11}\\ R_{12}\\ R_{13}\\ R_{21}\\ R_{22}\\ R_{23}\\ R_{31}\\ R_{32}\\ R_{33} \end{array}\right]$$

The matrix containing As for the set of *N* input vectors *A*_g_ and *H*_c_, is expressed as Equation (23):

(23) $$\left[\begin{array}{c} \text{const}\\ \vdots \\ \text{const} \end{array}\right]={\left[\begin{array}{l} A_{\text{g}x1}H_{\text{c}x1}\cdots A_{\text{g}xn}H_{\text{cx}n}\\ A_{\text{g}x1}H_{\text{c}y1}\cdots A_{\text{g}xn}H_{\text{c}yn}\\ A_{\text{g}x1}H_{\text{c}z1}\cdots A_{\text{g}xn}H_{\text{c}zn}\\ A_{\text{g}y1}H_{\text{c}x1}\cdots A_{\text{g}yn}H_{\text{c}xn}\\ A_{\text{g}y1}H_{\text{c}y1}\cdots A_{\text{g}yn}H_{\text{c}yn}\\ A_{\text{g}y1}H_{\text{c}z1}\cdots A_{\text{g}yn}H_{\text{c}zn}\\ A_{\text{g}z1}H_{\text{c}x1}\cdots A_{\text{g}zn}H_{\text{c}xn}\\ A_{\text{g}z1}H_{\text{c}y1}\cdots A_{\text{g}zn}H_{\text{c}yn}\\ A_{\text{g}z1}H_{\text{c}z1}\cdots A_{\text{g}zn}H_{\text{c}zn} \end{array}\right]}^{\text{T}}\cdot \left[\begin{array}{l} R_{11}\\ R_{12}\\ R_{13}\\ R_{21}\\ R_{22}\\ R_{23}\\ R_{31}\\ R_{32}\\ R_{33} \end{array}\right]$$

*Y* = φ · *x*.

The least squares solution *x* = (Φ^T^ · Φ)^−1^ · Φ^T^ · *Y* is derived by batch a least squares solution. In the calculation process according to the method proposed in [6] we find the problem that the condition number of matrix φ is larger, which decrease the estimation accuracy of the rotation matrix. Therefore, the Tikhonov method is adopted to obtain a better estimator.

### 4.2. Noise Suppression

A high rotation matrix accuracy plays an important role in further correcting the heading error, which is calibrated by the classical ellipsoid fitting method. The measurement noise, however, are inevitable and have influence on the estimator of rotation matrix *R*. The noise suppression methodology can reduce the sensitivity of the solution to measurement noise.

When the condition number of matrix φ in Equation (23) is very large, the matrix is approximately singular, or when observation data is inaccurate, the solution will be submerged by errors. The Tikhonov regularization method could solve the problem by adding other information about the solution, so as to identify a meaningful, stable solution [18]. The principle of adding information is that the 2-norm of the solution should be minimized. The regular solution defined by the regularization method is shown below [18]:

(24) $$\underset{x}{\min}{\left\|\varphi x-Y\right\|}_{2}^{2}+\lambda^{2}{\left\|Lx\right\|}_{2}^{2}$$

Where *L* is generally a unit matrix, λ is a regularization parameter that controls the sensitivity of Φ, and *Y* is the disturbance caused by the solution. The selection of λ mainly relies on experience to guarantee matrix $\left(\begin{array}{c} \varphi \\ \lambda L \end{array}\right)$ is of full column rank. A least squares solution is derived as the solution of Equation (24) is equivalent to the solution of Equation (25):

(25) $$\underset{x}{\min}{\left\|\left(\begin{array}{c} \varphi \\ \lambda L \end{array}\right)-\left(\begin{array}{c} Y\\ 0 \end{array}\right)\right\|}_{2}$$

Equation (25) can be derived from the QR (decompose a matrix into a orthogonal and an upper triangular matrix) orthogonal least square algorithm, which is equivalent to solving the following linear equations:

(26) $$(\varphi^{T}\varphi +\lambda^{2}L^{T}L)x=\varphi^{T}Y$$

***USV****^T^* is then obtained by singular value decomposition of the coefficient matrix (**φ***^T^***φ** + **λ^2^*L****^T^****L***). The coefficient matrix is a square matrix; matrix *U* is an orthogonal matrix and matrix *S* is a diagonal matrix containing the eigenvalues, *V* = *U*. Then *x* = (*USV^T^*)^−1^ φ*^T^* *y* = *V*^−^*^T^S*^−1^*U*^−1^φ*^T^* *y* and rotation matrix *R* will obtain by recasting matrix *x*. Though the noise suppression can reduce the influence of measurement noise, other methods are needed to ensure that *R* is close to a rotation matrix. For example, a quite large number of samples can improve the estimation accuracy. Another method is set a constraint *R^T^R* = I on matrix *R*, and try to minimize ‖*R* − *R*_m_‖ to obtain rotation matrix *R*. where *R*_m_ is recast by matrix *R, R* is the constrained optimization rotation matrix, its determinant is one. *R* is more close to the true rotation matrix *R*_0_ than *R*_m_. Introducing it into Equation (19) or the equation ***H*_e_** = ***R*** **·** ***H*_c_**, heading error will be calibrated completely.

## 5. Simulation Study

**Figure 3.**
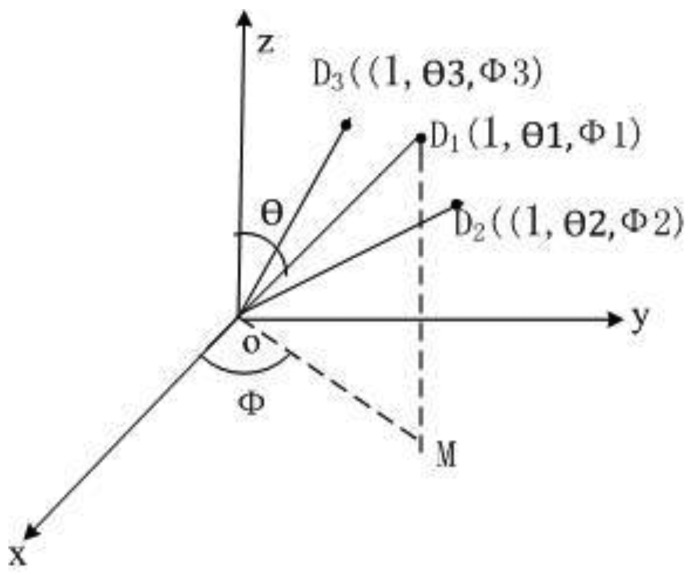
Collecting data according to the equivalent intersection angle principle.

**Figure 4.**
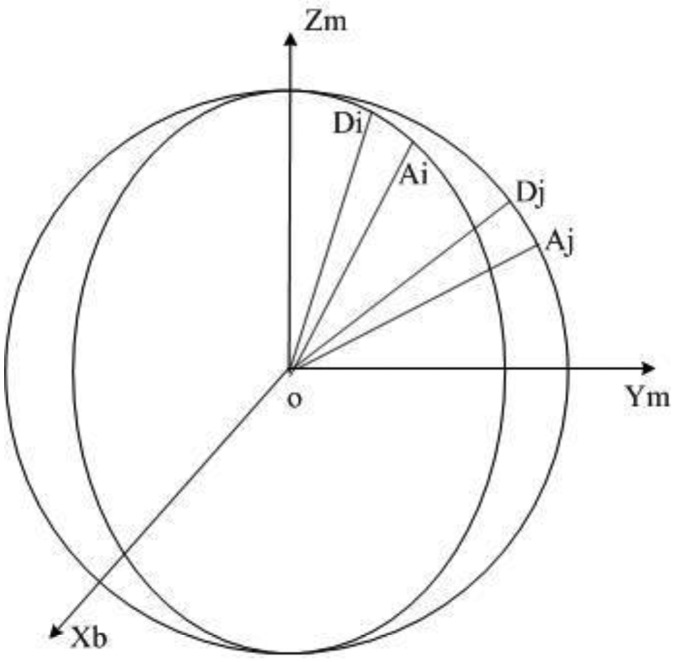
Constant intersection angle between the geomagnetic field vector and gravitation field vector.

During magnetometer calibration, to ensure the quality of the measurement data set, a proper uniform distribution of the data over all directions must be accomplished [19]. Firstly, 46 data points are collected for the simulation, which are evenly distributed on the sphere. According to the principles of the equivalent intersection angle, regarding the sphere center as the coordinate origin, and the radius of the sphere as one, the x-axis, y-axis and z-axis coordinates are collected in the *D*46_raw_ matrix, which is composed of 46 sample points. The collection method is shown in Figure 3, where the intersection angles ∠*D*_1_ *O D*_2_ and ∠*D*_1_ *O D*_3_ between any two adjacent data points are equal. Secondly, 46 data points are collected for the acceleration data, of which the magnitude is 9.8. As demonstrated in Figure 4, assuming that the acceleration vector and unit sphere intersects each other in *A_i_* and *A_j_*, ∠*D_i_* *O A_i_* is equivalent to ∠*D_j_* *O A_j_*.

Then assuming that error matrix $M=\left[\begin{array}{ccc} 1.06 & 0.02 & 0.03\\ 0.01 & 0.98 & 0.04\\ 0.02 & 0.05 & 1.1 \end{array}\right]$, zero deviation $H_{0}=\left[\begin{array}{c} 0.03\\ 0.02\\ 0.06 \end{array}\right]$, and hard iron error $H_{\text{p}}=\left[\begin{array}{c} 0.065\\ 0.02\\ 0.05 \end{array}\right]$, the output of the three-axis magnetometer influenced by the errors is *D*46_ellipse_ = *D*46_raw_ · M′, which is distributed on the disturbing ellipsoid. Then, the Gaussian white noise of *N*(0, 0.002^2^) is added into *D*46_ellipse_, where 0.002 is the standard deviation of the noise.

**Figure 5.**
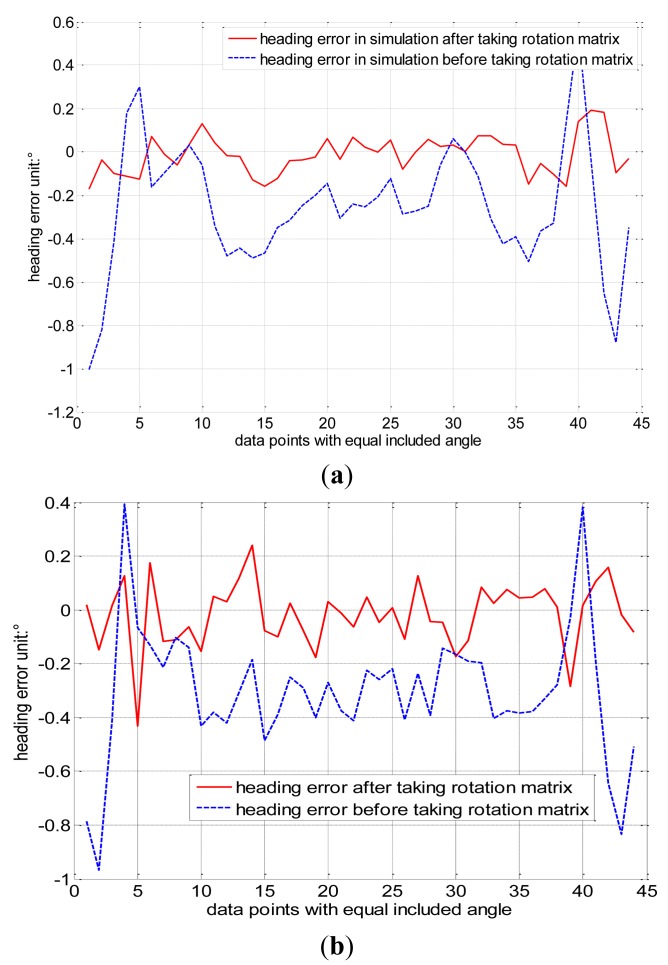

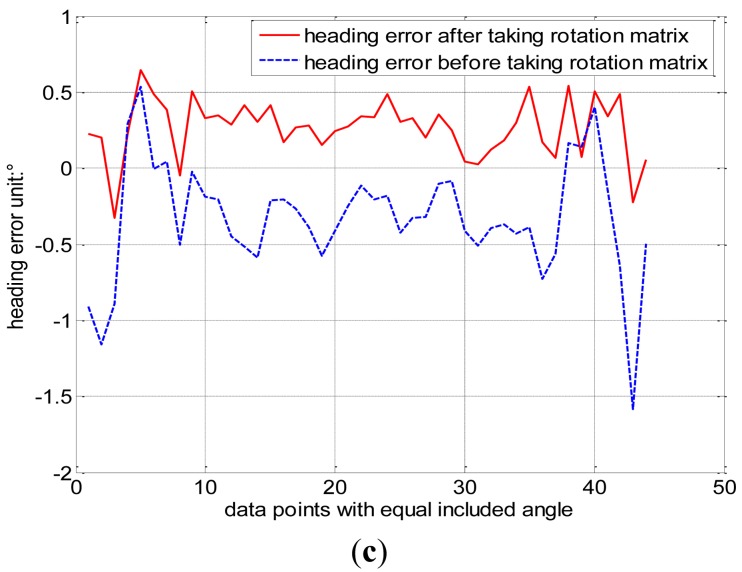
(a) Heading error when the standard deviation of the noise is 0.002; (b) Heading error when the standard deviation of the noise is 0.003; and (c) Heading error when the standard deviation of the noise is 0.005.

After determining the ellipsoid coefficient matrix, the preliminary calibration matrix *G* is derived by singular value decomposition. Regarding the heading as a reference, which is calculated from the original data set *D*46_raw_ that is free from the influence of the error matrix, the heading error calibrated by matrix *G* is shown in Figure 5a. The heading error is ±1° where the blue dashed line refers to the heading error curve after a preliminary calibration of matrix *G*, which is derived from the classical ellipsoid fitting. Because the ellipsoid model after fitting only contains nine independent parameters, it can’t determine the 12 error parameters in the error model completely. In other words, the classical ellipsoid fitting method can transform the ellipsoid into a sphere, where transformed sphere and the original sphere differ by an undetermined rotation matrix *R*. Consequently, this influences the accuracy of the heading calibration. The proposed constant intersection angle method can derive *R*.

The constant intersection angle method is not necessary for the additional calibration procedure mentioned in reference [11,12] and the coordinate transformation mentioned in reference [10]. Rotation matrix *R* can be derived from three-axis magnetometer and accelerometer data. The calibration matrix *G* derived from ellipsoid fitting coefficients is further corrected by rotation *R*, allowing us to improve the estimation of the calibration matrix *Q*. the heading error calibrated by matrix *Q* is lower than ±0.2°, which smaller than the heading error of ±1° when calibrated by classical ellipsoid fitting, shown as the red solid line in Figure 5a.

When the standard deviation of the Gaussian white noise is 0.003, and the magnitude of the simulation data is one, the heading error shown is shown in Figure 5b. The heading error calibrated by the constant intersection angle method decreases to ±0.4° from the value of ±1° when calibrated by the classical ellipsoid fitting. When the standard deviation of the noise is 0.005, the heading error is also shown in Figure 5c. The heading error decreases from ±1.2° to ±0.6° when calibrated by the constant intersection angle method. The error increases with the noise.

## 6. Experiments

### 6.1. Measurement Equipment and Measurement Spot

**Figure 6.**
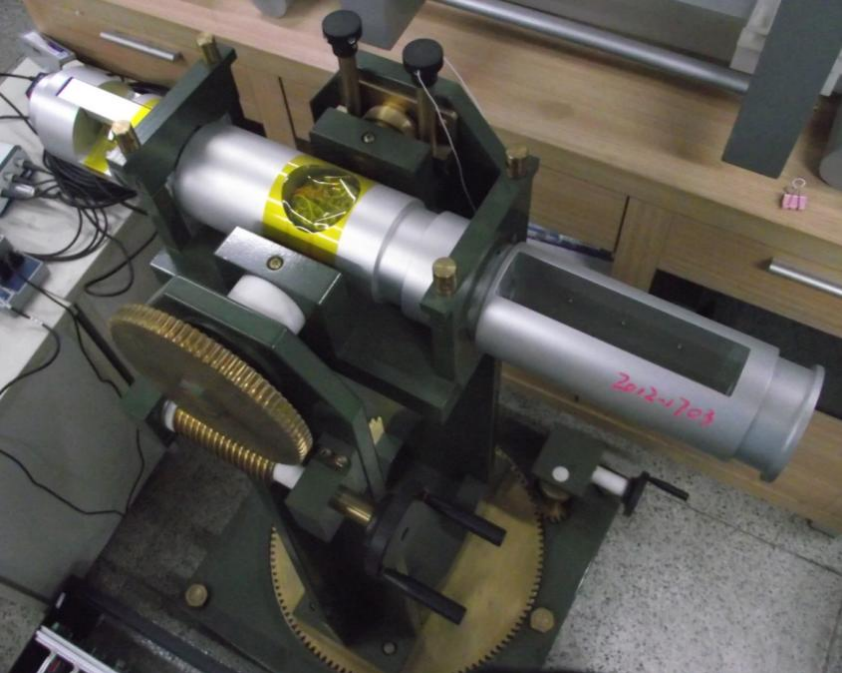
Nonmagnetic turntable.

In order to compare the calibration results between the classical ellipsoid fitting method and the constant intersection angle method proposed earlier, the nonmagnetic turntable experiment adopts a self-developed magnetic compass, which is mounted on the nonmagnetic turntable. The magnetic compass adopts a PIC24HJ128GP504 microprocessor (Microchip Technology, Chandler AZ, USA), equipped with a fluxgate sensor as the magnetic sensing element, and a Honeywell QA-T160 accelerometer (Honeywell Conglomerate Company, Morristown, NJ, USA) is used. The output heading angle of the optical–electrical encoder is regarded as the true heading reference, where the error is less than 3′. As shown in Figure 6, the magnetic compass is fixed on the nonmagnetic turntable. Where the body coordinate frame (*b*) takes the forward direction in the magnetic compass as the direction of *x*_b_-axis, taking the downward direction as the *z*_b_-axis, and setting the *y*_b_-axis in conformity with the left-hand rule, the navigation coordinate frame (*n*) takes a direction in the north-east-downward frame. It can be inferred from the body coordinate frame and navigation coordinate frame that the clockwise heading angle is positive, and the heading angle is 0° when the *x*_b_-axis points northward.

The experiment was performed in a Beijing suburb, where the magnetic environment in the vicinity of the magnetometer is better than that in the laboratory in the city. The principle of the constant intersection angle is adopted to ensure that the data points are distributed evenly on the sphere. The data collection is composed of 46 sets of data spread on the *x*-axis, *y*-axis and *z*-axis, collected as follows. To begin, where the magnetic compass is fixed on the nonmagnetic turntable, and the roll angle is 0°, and pitch angle is 60° and −60°, respectively, the nonmagnetic turntable is rotated clockwise such that the magnetic compass *x*_b_-axis can intersect with the north direction at angles of 6°, 66°, 126°, 186°, 246° and 306°; Where the roll angle is 0°, and the pitch angle is respectively 60° and −60°, the nonmagnetic turntable is rotated clockwise such that the magnetometer *x*_b_-axis can intersect with the north direction at angles of 6°, 42°, 78°, 114°, 150°, 186°, 222°, 258°, 294° and 330°. Where both the roll angle and the pitch angle are 0°, the nonmagnetic turntable is rotated clockwise such that the magnetometer *x*_b_-axis can intersect with the north direction at angles of 6°, 36°, 66°, 96°, 126°, 156°, 186°, 216°, 246°, 276°, 306°, and 336°. Where the roll angle is 0°, and the pitch angle is 90° and −90°, the *x*_b_-axis is vertically upward and downward, respectively. The accelerometer data should be collected simultaneously at the same 46 positions, because the method is attitude-dependent.

Then, magnetometer preliminary calibration can be conducted through the ellipsoid assumption method. The heading angle ψ could be is derived Equation (27). It should be noted that magnetic field data in the body coordinate frame $\left(H_{x}^{b},H_{y}^{b},H_{z}^{b}\right)$ should be transformed to the navigation coordinate frame $\left(H_{z}^{n},H_{y}^{n},H_{z}^{n}\right)$. The transformation matrix is shown in Equation (28):

(27) $$\psi =\{\begin{array}{lll} \alpha & & \left(H_{y}^{n}\geq 0,H_{x}^{n}>0\right)\\ & \pi +\alpha & \left(H_{x}^{n}<0\right)\\ 2\pi +\alpha & & \left(H_{y}^{n}<0,H_{x}^{n}>0\right)\\ & \frac{\pi }{2}(H_{y}^{n}>0,H_{x}^{n}=0) & \\ & \frac{3\pi }{2}(H_{y}^{n}<0,H_{x}^{n}=0) & \end{array}$$

where $\alpha =\tan^{-1}(H_{y}^{n}/H_{x}^{n})$.

(28) $$\left[\begin{array}{c} H_{x}^{n}\\ H_{y}^{n}\\ H_{z}^{n} \end{array}\right]=\left[\begin{array}{ccc} \cos\theta & \sin\gamma \cos\theta & -\sin\gamma \cos\theta \\ 0 & \cos\theta & \sin\theta \\ \sin\gamma & -\sin\theta \cos\gamma & \cos\gamma \cos\theta \end{array}\right]\cdot \left[\begin{array}{c} H_{x}^{b}\\ H_{y}^{b}\\ H_{z}^{b} \end{array}\right]$$

θ and γ respectively refer to the pitch angle and roll angle, which are determined by the three-axis accelerations *g_x_, g_y_*, and *g_z_*, when either angle is not zero and where $g=\sqrt{g_{x}^{2}+g_{y}^{2}+g_{z}^{2}}$:

(29) $$\theta =-\sin^{-1}{(}^{g_{x}}{/}_{g})$$

(30) $$\gamma =-\sin^{-1}\left(\overset{g_{y}}{\;}/\underset{g}{\;}\right)$$

### 6.2. Accelerometer Calibration

As a type of inertial sensor, accelerometers are reliable, independent, and immune to environmental impacts. The primary errors of accelerometers are bias, sensitivity error, and non-orthogonal error. Such errors are similar to the magnetometer errors, as observed in Equation (1–3). The following equation describes the accelerometer observation:

(31) $$g_{\text{m}}=k_{\text{s}}\cdot k_{\text{nor}}\cdot g_{\text{e}}+g_{0}=C\cdot g_{\text{e}}+g_{0}$$

where, *g*_0_ is the bias of accelerometer, *g*_e_ is the actual acceleration of gravity, and $C=\left[\begin{array}{ccc} S_{x} & 0 & \alpha S_{x}\\ \beta S_{y} & S_{y} & \gamma S_{y}\\ 0 & 0 & S_{z} \end{array}\right]$ is derived from Equation (1) and Equation (3). Generally, the non-orthogonal angle of a three-axis accelerometer provided by the manufacturer is less than 0.5°. For calculation purposes, the trigonometric functions of Equation (3) can be simplified. The actual acceleration of gravity is derived from Equation (31) as follows:

(32) $$g_{\text{e}}=C^{-1}\cdot \left(g_{\text{m}}-g_{0}\right)$$

**Figure 7.**
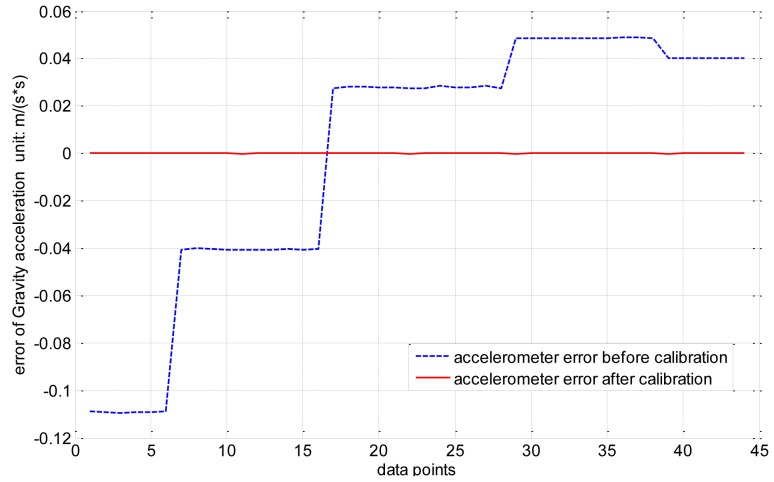
The accelerometer error before calibration and after calibration.

The output trajectory of the three-axis accelerometer accords with the ellipsoid hypothesis as well. There are nine unknown parameters consisting of three sensitivity errors, three non-orthogonal error and three bias. Thus, the error parameters of the accelerometer can all be derived from the coefficients of the ellipsoid equation. The experiment is performed on the turntable shown in Figure 6 by mounting the magnetic compass on the aligned turntable, aligning the body frame of the magnetic compass with the turntable by using the location pins, and then rotating the turntable to different angular positions. The results in Figure 7 show that the absolute error declines from 0.1582 m/s^2^ to 4.6323 × e^−4^ m/s^2^, and the mean square root error drops from 0.0545 m/s^2^ to 1.0358 × e^−4^ m/s^2^; thus, the maximum error of the accelerometer can be reduced by a factor of approximately 1,000.

### 6.3. Experimental Result

**Figure 8.**
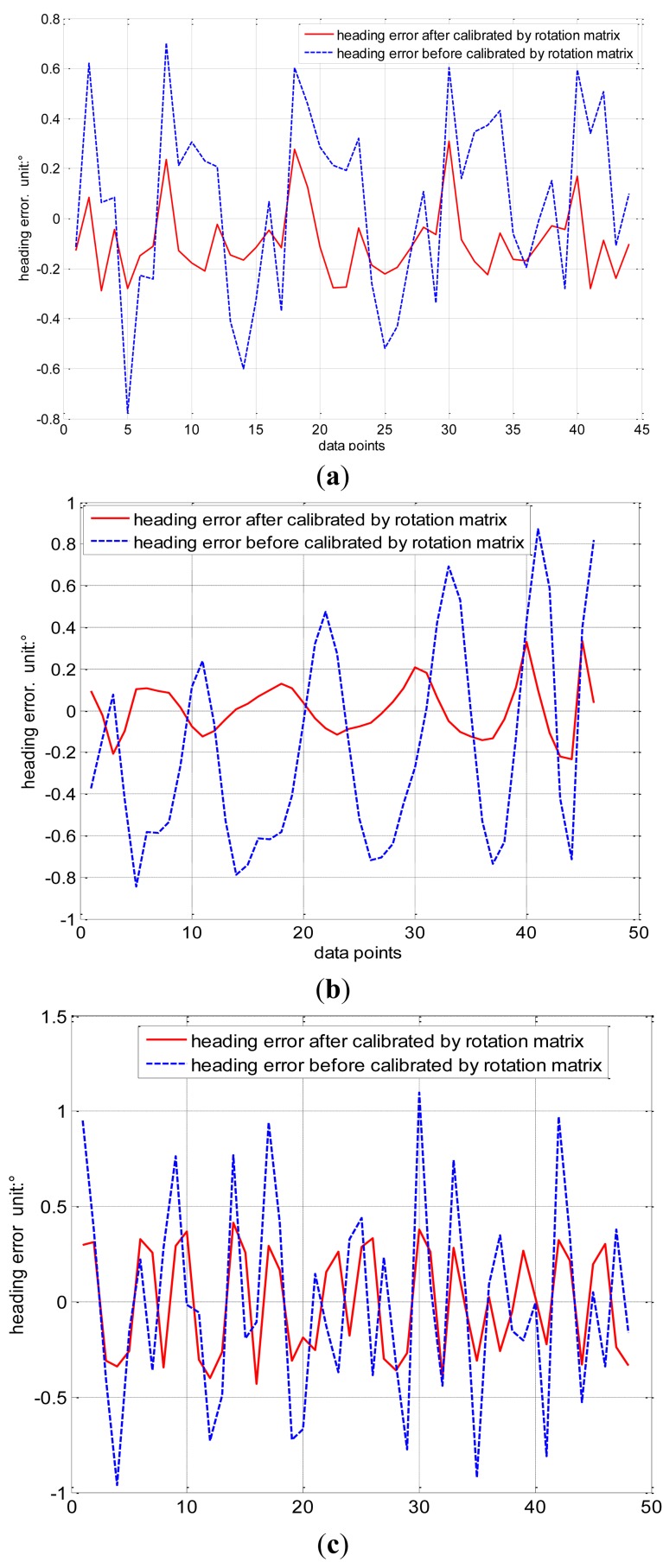
(**a**) Experiment results comparing heading error in Beijing suburbs; (**b**) experimental results comparing heading error in laboratory in the city; and (**c**) more experimental results comparing heading error in the laboratory in the city.

The calibration matrix *G* of Equation (18) is derived from the ellipsoid coefficient matrix by eigen-value decomposition and singular value decomposition in the classical ellipsoid fitting method. Then *G* is introduced into Equation (20) to calibrate the measurement of the geomagnetic field *H*_m_ and obtain *H*_c_ calibrated by the classical ellipsoid fitting method. However, calibration matrix *G* is not the actual calibration matrix, which should be multiplied by the rotation matrix *R* mentioned earlier. *R* is obtained using the constant intersection angle method to further improve the accuracy of magnetic sensor. This method can be used to other type of three-axis inertia sensor including misalignment error. Then, the complete calibration geomagnetic field *H*_e_ = *R* · *H*_c_ is obtained. The heading error is shown in Figure 8a. The red solid line refers to the heading error calibrated by the constant intersection angle method, which is less than ±0.3°, and the blue dashed line refers to the heading error calibrated by the classical ellipsoid fitting method, which is within the range of ±0.8°.

Figures 8b,c are the results of two other experiments conducted in the laboratory in the city, where the red solid line refers to the heading error calibrated by the constant intersection angle method, and the blue dashed line refers to the heading error calibrated by the classical ellipsoid fitting method. The maximum heading errors calibrated by the constant intersection angle method are ±0.35° in Figure 8b and ±0.4° in Figure 8c. The maximum heading errors calibrated by the classical ellipsoid fitting method are approximately ±0.9° in Figure 8b and ±1.1° in Figure 8c. This shows that the constant intersection angle method has similar calibration performance in the laboratory in the city. The heading errors after calibration are slightly larger because magnetic perturbations are larger in the city.

**Figure 9.**
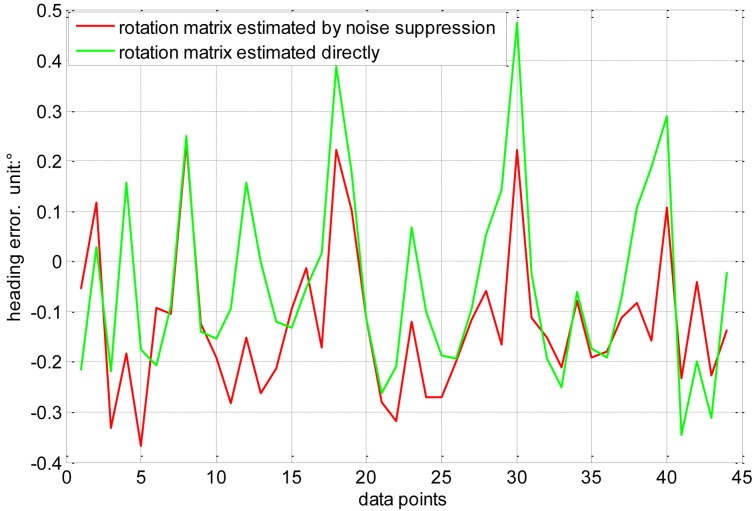
Comparison experiment about the noise suppression methodology.

As mentioned in Section 4.2, the accuracy of rotation matrix *R* affects the heading error directly. The measurement noises, however, are inevitable and have influence on the estimator of rotation matrix *R*. The noise suppression methodology can reduce the sensitivity of the solution to measurement noise, and the constrained optimization (set a constraint *R^T^R* = *I* on matrix *R*) can further improve the estimation accuracy of *R*. In order to verify the effect of this methodology, we conduct a comparison experiment shown in Figure 9.

The green line refers to the heading error that the rotation matrix *R* is estimated directly by the measurement data; the red line is the heading error that the estimation of *R* is adopted the noise suppression methodology. It can be seen from Figure 9 that the heading error decrease from ±0.48° to ±0.32°.

## 7. Conclusions

This study thoroughly analyses the three-axis magnetometer measurement errors and establishes a complete error model including 12 independent parameters, which is more universal and conforms to the ellipsoid restriction. However, the calibration matrix derived from the ellipsoid coefficient matrix by a different matrix decomposition method is not unique, and there exists an unknown rotation matrix *R* between them [5]. A constant intersection angle method (angles between geomagnetic vector and gravitational vector are fixed in the same location) is proposed to estimate *R*. The geomagnetic field vector and heading error are further corrected by *R*. The simulation experiment indicates that the heading error declines from ±1° when calibrated by the classical ellipsoid fitting method to ±0.2° when calibrated by the constant intersection angle method, and the signal-to-noise-ratio is 50 dB. The actual experiment demonstrates that the heading error is further corrected by the constant intersection angle method decreases from the value of ±0.8° when calibrated by the classical ellipsoid fitting method to a value of ±0.3°. The method proposed is convenient and practical, as it is free from any additional calibration procedure mentioned in references [11,12] and the coordinate transformation mentioned in reference [10]. In addition, the noise suppression methodology can reduce the sensitivity of the solution to measurement noise and obtain higher accurate estimator of the rotation matrix than reference [6].
